# “We just have to work with what we’ve got”: a qualitative analysis of contextual challenges in facilities and resources for pupil physical activity in English primary schools

**DOI:** 10.1186/s12889-025-21895-1

**Published:** 2025-02-21

**Authors:** Danielle House, Robert Walker, Lydia Emm-Collison, Simona Kent-Saisch, Ruth Salway, Alice Porter, Frank de Vocht, Russell Jago

**Affiliations:** 1 Population Health Sciences, Bristol Medical School, University of Bristol, Bristol, UK; 2 Centre for Exercise, Nutrition & Health Sciences, School for Policy Studies, University of Bristol, Bristol, UK; 3 NIHR Bristol Biomedical Research Centre, University Hospitals Bristol and Weston NHS Foundation Trust and University of Bristol, Bristol, UK; 4 National Institute for Health and Care Research Applied Research Collaboration West (NIHR ARC West), The National Institute for Health and Care Research, University Hospitals Bristol and Weston NHS Foundation Trust and University of Bristol, Bristol, UK

**Keywords:** Physical activity, Children, School-based, Primary schools, Context, Tailored-intervention, Built environment

## Abstract

**Background:**

State primary schools present an opportunity to support children’s physical activity equitably, however, many school-based physical activity interventions have been unsuccessful. Many interventions have focused on school built environments to increase or measure the physical infrastructure a school has. Yet literature suggests that broader social and institutional factors, such as school policies and staffing, are equally crucial for supporting pupil’s physical activity. The aim of this study is to qualitatively explore and assess primary school staff perspectives on the role of school facilities and resources in supporting pupil physical activity and the factors that impact their use.

**Methods:**

We conducted 33 semi-structured interviews with state primary school staff in the Bristol area, Southwest England, between November 2023 and January 2024. Staff roles included headteacher/principal (*n* = 5), deputy headteacher/principal (*n* = 6), class teacher (*n* = 7), teaching assistant (*n* = 2), Physical Education (PE) subject lead (*n* = 8), dedicated PE teachers (*n* = 4), and Parent Teacher Association chair (*n* = 1). Staff were recruited from 19 purposively-sampled schools with a range of school sizes, locations, and sociodemographic characteristics. Reflexive thematic analysis was used.

**Results:**

Five themes were generated related to how school facilities and resources impact pupil physical activity: 1) Facilities and resources create potential for pupil physical activity, 2) A social context supportive of physical activity is needed to maximise this potential, 3) Logistical challenges undermine the potential of facilities and resources, 4) The potential of facilities and resources change throughout the school year, and 5) Facilities and resources are not always fit for purpose.

**Conclusion:**

The presence of facilities or resources alone does not determine pupil physical activity at primary school. Factors such as the social context of the school, logistical and timetabling challenges, seasonality, and how fit for purpose facilities are for that school, are important factors in school staff and pupils’ use and management of these facilities, and whether their potential is maximised or undermined. Future research and policy must broaden the focus from the presence of facilities and resources for physical activity, to include a focus on how these are maintained and engaged with in a school’s specific context.

**Supplementary Information:**

The online version contains supplementary material available at 10.1186/s12889-025-21895-1.

## Introduction

Physical activity is important to current and future children’s health and wellbeing globally [1–3]. The World Health Organization and UK Chief Medical Officers recommend that children engage in an average of one hour of moderate to vigorous intensity physical activity (MVPA) per day, accumulated across a week [4, 5]. In England, these recommendations are not met, where, despite returning to pre-COVID-19 levels, only 41% of 10–11-year-old children meet the advised levels [6]. There is evidence the COVID-19 pandemic has widened pre-existing physical activity inequalities [7–11]. It is therefore important we find strategies to support and increase children’s physical activity.

To date, strategies to increase children’s physical activity in England have often focused on state primary schools (publicly funded schools that provide free education to children ages 4–11, in which approximately 93% of children are enrolled [12–15]. Schools provide an opportunity to deliver interventions equitably to large numbers of children. The World Health Organization’s Health Promoting Schools framework advocates for integrating health promotion, including physical activity, across all aspects of school life [16], which has been shown to have positive average impacts on physical activity [17]. This has aligned with a shift towards whole-school physical activity promotion being implemented in international settings that involve integrating physical activity into the entire school environment and culture, ensuring it becomes a core part of the daily lives of students and staff [18]. An example of such a framework in the UK includes the Creating Active Schools framework [19].

In a recent scoping review, we identified 11 physical activity opportunities targeted by interventions implemented in European primary schools, most frequently targeting physical education (21%), active and outdoor learning (16%), active breaks (15%), and school-level environment (12%) [20]. However, to date, the majority of interventions have not yielded significant improvements in children’s MVPA [21–29]. Elsewhere we have argued that this failure is partly attributable to the “one size fits all” approach that characterises the majority of physical activity interventions, whereby an intervention is delivered identically across diverse schools and populations, and does not account for significant variations in primary school contexts [29]. By primary school context, we mean the set of characteristics that shape the school, including its local environment, the demographic profile of the pupils, the facilities available, the skill and attitudes of staff, school priorities, and the interests of the children [29]. These contextual differences between schools impact upon how interventions are implemented, how novel the intervention is, how suitable it is for that school, and in turn how effective it may be. In a related paper using the same data set as this present analysis, we explored the role of school social context in pupils’ physical activity. This analysis identified six key social contextual factors influencing pupil physical activity (two upstream factors and four school-level factors). Upstream factors included: 1) Regulatory systems and pressures and 2) limited Physical Education (PE) training during teacher training. School-level social factors included: a) Senior Leadership Team (SLT) (*e.g.* headteacher/principle or deputy headteacher/principle) personal values and priorities; b) pupil needs; c) passionate individual(s); and d) staff confidence and capability (Walker, et al.: The complexity of promoting physical activity in English state primary schools: an in-depth qualitative analysis of the role of social context, Under review).

Alongside the social context, the physical or built environment constitutes another important facet of primary school context. To date, much academic work that has examined the relationship between school physical environments and pupil physical activity has sought to identify the key physical features of a school that influence or support pupil physical activity. This work has, in the main, been undertaken via school audits or the evaluation of school interventions and has focused predominantly on the school playground [30–37]. Although a recent scoping review suggested school playground markings increased pupil physical activity [31], findings across this literature are inconsistent regarding associations between increasing particular school facilities and MVPA or physical activity behaviour [30, 32, 36–38].

Literature exploring school built environments and pupil physical activity suggests that broader social and institutional factors, such as school policies and staffing, are equally crucial for supporting pupil’s physical activity [34, 36, 37]. It is therefore important to examine the experience of delivering and supporting pupil physical activity in primary schools to shed light on these broader factors and improve our understanding of how any school facilities or resources are used, managed, and engaged with. Understanding the contextual nuances of schools through qualitative insights may also address gaps in prior interventions that failed due to a lack of contextual tailoring. The aim of this study is to qualitatively explore and assess primary school staff perspectives on the role of school facilities and resources in supporting pupil physical activity and the factors that impact their use.

## Methods

### Participants and procedure

The Physical Activity via a School Specific PORTfolio (PASSPORT) project [39] is designing a school-context specific intervention for children’s physical activity in English primary schools. Key to this work is the design of a tool for schools to use to audit their context and then select intervention components that are suitable for their school [29]. This present study draws on qualitative data collected as part of the formative work for PASSPORT. Data were collected via two sets of semi-structured interviews with state primary school staff. The first interviews explored the elements of school context that impact pupil physical activity and the second explored the feasibility and acceptability of a portfolio intervention design. Important data related to school context was discussed in both sets of interviews, therefore both datasets were included in this analysis.

A diverse set of primary schools in the wider Bristol area, England, were purposively sampled to ensure a range of school contexts were included in the study. Publicly available government and local authority data was used to track and target recruitment. School postcode (to ensure a geographical spread) and size (number of pupils) [40], the percentage of pupils eligible for free school meals ([41] FSM, a UK government scheme to provide free meals at school to children from low income families [42]), and school area Index of Multiple Deprivation decile, derived from school postcode (IMD [43]), were all used in recruitment. We used the percentage of people that belong to a black, Asian, mixed, or other ethnic group in the Lower Layer Super Output Area (LSOA [44]) the school was situated in [45] to capture diverse cultural and religious school contexts (although we recognise the significant shortcomings of the classification [46]). Although recruitment efforts targeted diverse schools, those with a strong emphasis on physical activity may have been more inclined to participate, potentially limiting the representation of less active schools. In total, staff from 19 state primary schools were recruited to the study, and the characteristics of these schools are reported in Table 1. Overall, diverse socioeconomic, geographic, and schools within minoritised ethnic communities participated in this study, providing important insight for understanding school context. Across these 19 schools, 33 interviews were conducted (18 using the context interview guide and 15 using the feasibility interview guide) with 32 members of school staff and the wider school community (one participant undertook two interviews; one with each topic guide). One interview was conducted in 11 of the 19 schools, two interviews in three schools, three interviews in four schools, and four interviews in one school. The research team reflected on ongoing participant and school recruitment and purposively sampled participants to include a range of roles in each of the two sets of interviews, ensuring the sample size was adequate and diverse. The team also reflected on the emerging data to consider whether we were meeting the research aims [47]. Participant roles can be seen in Table 2. Interviews were conducted between November 2023 and January 2024 by RW, DH, and AP, and lasted between 22 and 49 min. Six interviews were conducted in person, while the remaining 27 took place via MS Teams. Interviews were audio recorded. The training and experience of the interviewers are as follows:

RW has completed postgraduate-level training in interviewing and possesses seven years of experience in qualitative research. He has led qualitative components of three interdisciplinary research projects, utilising various methods with both children and adults, conducting over 100 interviews.

DH has specialised in qualitative methods, including interviewing, throughout her career, spanning 12 years. She has received training in interviewing during her undergraduate and postgraduate education and has conducted interviews for approximately 10 academic and practice-based research projects.

AP has undertaken postgraduate-level interviewing training through the Bristol Medical School, University of Bristol, and the Social Research Association, UK. With seven years of research experience, AP has conducted interviews for four research projects, totalling over 60 interviews.

The protocol for this study was published on the Open Science Framework [48]. This study was approved by the University of Bristol, Faculty of Health Science Research Ethics Committee (FREC Ref 15866). Informed consent was obtained for all participants, who received a £25 gift voucher as recompense of their time.

**Table 1:** School characteristics

**School characteristics**		
* Urban/rural classifications*		
Urban		10 (52.6%)
Suburban		8 (42.1%)
Rural		1 (5.3%)
* Free School Meal*^*a*^* %*		
Below national average (23.8%)		10 (52.6%)
Above national average (23.8%)		9 (47.4%)
* School postcode Index of Multiple Deprivation*^*b*^		
1-3		9 (47.4%)
4-6		6 (31.6%)
7-10		4 (21.1%)
* School Lower Layer Super Output Area*^*c*^* people that belong to a black, Asian, mixed, or other ethnic group*^*d*^* %*		
Below national average (18.3%)		8 (42.1%)
Above national average (18.3%)		10 (52.6%)
No data^e^		1 (5.3%)
* Number of pupils in school*		
0-200		1 (5.3%)
201-400		6 (31.6%)
401-600		7 (36.8%)
601+		4 (21.1%)
No data^f^		1 (5.3%)
* School management*		
Local authority maintained (LAM)		7 (36.8%)
Multi-academy trust (MAT)		12 (63.2%)

^a^national average was 23.8% in 2023 [36]

^b^a measure of area deprivation, with 10 being least deprived and 1 being most deprived [38]

^c^a Census-based geographic unit for the reporting of small area statistics in England and Wales of roughly 1000-3000 people [39]

^d^defined as all ethnic groups except white ethnic groups [40]

^e^No data for School Lower Layer Super Output Area reflected incomplete demographic records for one school

^f^No data for number of pupils reflected incomplete demographic records for one school

**Table 2:** Participant roles at school

Participant roles^a^	n
Headteacher/Principal	5
Deputy Headteacher/Principal	6
Class Teacher	7
Teaching Assistant	2
Physical Education Lead	8
Dedicated Physical Education Teacher	4
Parent Teacher Association Chair	1

^a^Participants often had more than one of these roles in a school, for example, both a class teacher and the PE lead. Participants with multiple roles are prioritised by PE, then SLT (Headteacher/Principal and Deputy Headteacher/Principal), then class teacher roles

### Study materials

Topic guides for both sets of interviews (Supplementary Files 1 and 2) were developed by the research team. Patient and Public Involvement (PPI) input from a Key Stage 2 (children aged 7-11) state primary school teacher was used to validate questions, ensuring they were appropriate for the target population. The first guide focused on opportunities for physical activity in school and the ‘real life’ challenges to this, to highlight contextual factors between schools. The second guide explored initial design ideas for a tailored intervention and stepped-wedge evaluation, including the contextual factors that may lead to implementation challenges.

### Data analysis

This study is based in interpretive research design which adopts a constructivist ontological stance that views the world as socially constructed, and an epistemological stance that focuses on understanding the subjective meanings and experiences of individuals within their social context [49]. This study used reflexive thematic analysis [50] recognising the researcher’s interpretive analysis of the dataset. The analysis was supported by NVivo 13 [51] and involved six stages, undertaken by DH unless specified otherwise: 1) data familiarisation (reading the transcripts), 2) the independent coding of two transcripts from each set of interviews (four in total) by DH, RW, and AP to compare codes and discuss interpretations, 3) initial inductive coding of the full dataset iteratively with codes refined during the process, 4) secondary coding of the dataset to ensure coding was consistently applied, 5) theme generation by interpreting the coded dataset, and 6) refinement of the themes. To ensure reflexivity, the analysis process included regular team discussions and coding comparisons to promote higher quality and more nuanced interpretations of data.

## Results

**Fig. 1:**
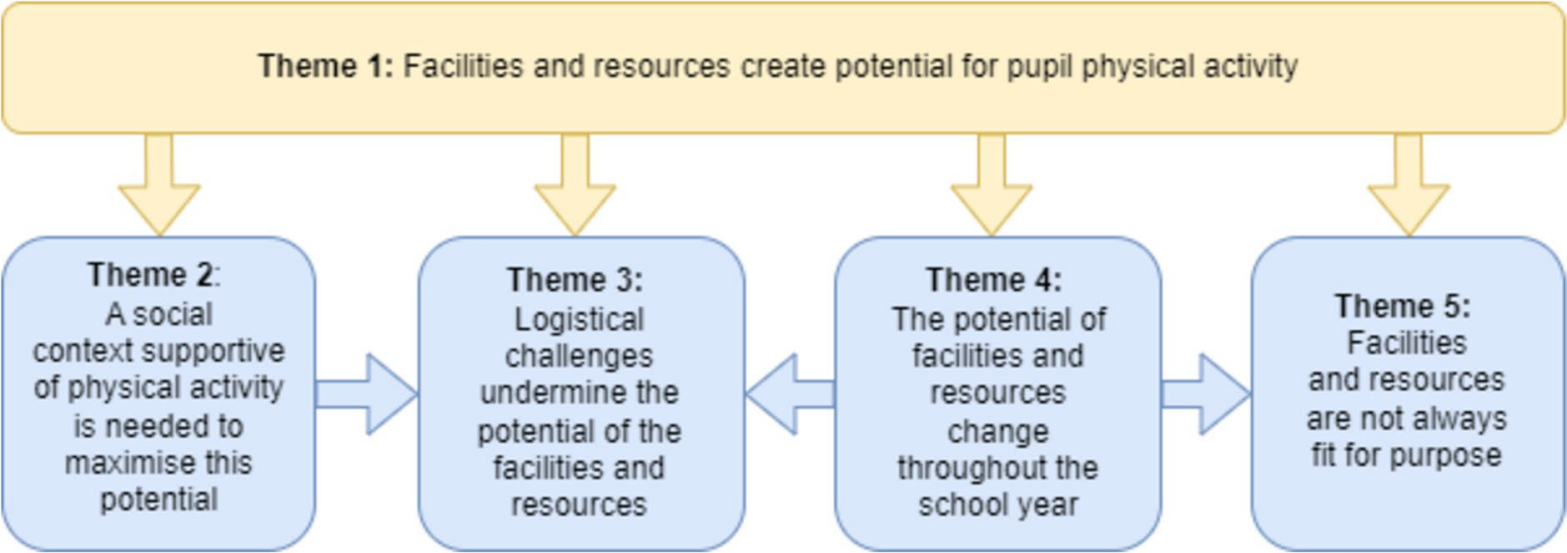
Diagram of key findings

Five themes were generated related to the contextual factors that influence the potential of school facilities and resources to support pupil physical activity. These are: 1) Facilities and resources create potential for pupil physical activity, 2) A social context supportive of physical activity is needed to maximise this potential, 3) Logistical challenges undermine the potential of facilities and resources, 4) The potential of facilities and resources change throughout the school year, and 5) Facilities and resources are not always fit for purpose. Table 3 provides an overview of the themes generated and a brief description, and Fig. 1 shows the theme relationship.

**Table 3:** Theme overview

Theme name	Description
1) Facilities and resources create potential for pupil physical activity	This theme explores how the facilities and resources a school has access to shapes the potential for pupil physical activity, but alone is not enough to determine pupil physical activity. The potential that is created with the existence (or not) of facilities and resources can be undermined or maximised, and is explored in the following four themes.
2) A social context supportive of physical activity is needed to maximise this potential	This theme identifies how a school’s social context, i.e., staff experience, priorities, and values, particularly the Senior Leadership Team (SLT) shapes how facilities and resources are maximised for pupil physical activity. The maintenance of or investment in facilities and resources can be deprioritised, or innovative solutions can maximise potential.
3) Logistical challenges undermine the potential of the facilities and resources	This theme demonstrates how logistical challenges can undermine the potential of facilities and resources. While schools may have facilities or resources, competing priorities often limit their effective use.
4) The potential of facilities and resources change throughout the school year	This theme explores the impact of seasonality on the potential of facilities and resources for pupil physical activity, both in terms of seasonal weather but also times in the school year when facilities are under increased pressure.
5) Facilities and resources are not always fit for purpose	This final theme describes how the facilities and resources a school has are not always suitable for supporting pupil physical activity, due to issues such as design or lack of adaptability to a specific school context.

Engagement with our data highlighted that the language used by participants varied across contexts. We therefore generated the following definitions that we felt best captured the meanings within our data. By facilities we mean the spaces in the built environment of a school (or elsewhere, if schools access offsite facilities) that are used for pupil PE and physical activity, such as playgrounds or indoor halls. By resources we are referring to the additional elements needed to undertake PE and physical activity in these facilities, namely, equipment, staff (including external provision), and teaching materials. The inclusion of not just facilities (built environment) but the resources needed to enliven these was data driven, as the relatedness of these was clear throughout the interviews. Furthermore, in this analysis we include discussion of both PE, as a foundational subject in a school’s curriculum, and broader physical activity, the opportunities across a school day for children to be active. We recognise the limitation of combining these and do not see these as interchangeable. However, the terms were often used interchangeably by participants as PE leads or teachers often have responsibility for broader pupil physical activity, e.g. active clubs. Within the aim of this study to examine the role of school facilities and resources in pupil physical activity, discussion of PE and physical activity was present across the data.

### Theme 1: facilities and resources create potential for pupil physical activity

Across the interviews, participants explained how the facilities and resources schools have access to created potential for their PE teaching and broader physical activity provision for pupils. Several schools recognised how their facilities, such as indoor halls or outdoor space, supported the quantity and quality of their provision and enabled them to support pupil activity. For example, one school expressed that they feel lucky to have the facilities to ensure children have spaces where they can be active:

*“So we're quite lucky in the school. So I said, yeah, we've got two halls, they're great. We lost a bit of space because we built the Year 5 classes when the school went bigger on the lower playground. But we still have an upper playground and we've got something called a MUGA, which is a multi-use games space, and then we've got a large field.”(Participant 10, Class Teacher, school FSM% 9.8, IMD 10)*

Conversely, schools reported the challenge of a lack of facilities and resources, and how greater availability of, for example, indoor space for PE or sports equipment to engage a greater number of pupils or provide broader opportunities, would benefit pupils and positively impact what the schools can do, demonstrating the related nature of facilities and resources. As one participant explains:

*“It’s space. If we could have a bigger shed to house more equipment, we’d have more equipment. The equipment [now] is the bare essentials, the bare minimum. Yes, I think space is a huge issue.” (Participant 12, PE Lead, school FSM% 10.8, IMD 8*

This was also apparent in terms of staff as a resource to support physical activity opportunities, where several schools commented that they were able to provide opportunities because they had a large teaching staff body, or conversely that they were a smaller school and so unable to staff many opportunities such as active clubs and or taking children to competitions.

*“We’re quite lucky in this school, obviously it being large. […] Because every teacher is encouraged to do a club. So obviously, having a large teaching force, we can therefore offer loads of clubs.” (Participant 10, Class Teacher, school FSM% 9.8, IMD 10)*

However, across the interviews, participants gave nuanced insights into how facilities and resources are used, maintained, and engaged with, which undermined or maximised the potential of these to support pupils to be active, demonstrating the complex relationship between school facilities and pupil physical activity. The following four themes explore these aspects.

### Theme 2: a social context supportive of physical activity is needed to maximise this potential

This theme identifies a connection between how a schools’ social context, i.e. their staff experience and values, their pupils, and their priorities (Walker, et al.: The complexity of promoting physical activity in English state primary schools: an in-depth qualitative analysis of the role of social context, Under review), shapes how facilities and resources are maximised for pupil physical activity. Participants expressed a sense that a school has to make a concerted effort to maximise the facilities and resources they have for physical activity, and that if a school did not prioritise this then facilities could be under-utilised. The ‘buy in’ of staff at all levels – Senior Leadership Team (SLT), PE teachers/leads, class teachers, and lunch time supervisors – to maximise the potential of facilities, was explicitly noted by many participants as more important than the simple existence of the facilities (Theme 1).

*“I think regardless of how much space we’ve got, which we are very fortunate to have, without that buy-in, that space had might as well be dead land. We’d might as well sell it and let developers build on it, kind of thing, because if you haven’t got that buy in from everyone, it doesn’t work.” (Participant 11, Class Teacher and SLT, school FSM% 31.6, IMD 2)*

However, the role of the SLT specifically in this buy in was clear, due to their role in setting school priorities and strategies (Walker, et al.: The complexity of promoting physical activity in English state primary schools: an in-depth qualitative analysis of the role of social context, Under review). For example, SLT could maximise or undermine the potential of facilities and resources via decisions around maintenance (particularly in a context of squeezed school finances), training staff to utilise facilities and resources to support children to engage with these, staff resourcing for break times, or incentivising staff to run extra-curricular clubs (both active and others).

*“[the school] don’t really have [a site team] at the moment. So, that has an immediate knock-on with, at the moment, leaves that are everywhere. So, using the MUGA [multi-use games area] can be unsafe, because it can be slippery. […] And if they don’t have anyone to clean it, the kids can’t use it. There’s a trim trail [a series of play equipment] at the top, it’s out of bounds quite often, just with a piece of tape around it, because it can’t be maintained if there’s no site team.” (Participant 1, Parent Teacher Association Chair, school FSM% 36.6, IMD 2)*

*“There are always bikes, scooters, skateboards and things like that [in the playground]. But there’s not always football on anymore, because you need one member of staff to run it on the football pitch. […] There used to be SLT [available] and sometimes there isn’t a member to do that anymore.” (Participant 4, Teaching Assistant, school FSM% 48.0, IMD 1)*

However, across the interviews, primary school staff also provided examples of how they worked to overcome limitations of their facilities and resources by finding innovative solutions and making adaptations to maximise the physical activity opportunities and quality for their pupils. Some schools had adapted their school uniform policy so pupils arrive dressed for PE on PE days, and others had taken on a universal active uniform. Those with an active uniform explained that children could then be active throughout the day, maximising breaktimes for physical activity, as they are always appropriately dressed. Other schools incentivised pupils to maintain sports and play equipment when there was no staff capacity to do so.

*“…we’ve got PE leaders [within Year 6 classes]. […] once a week they go outside, do a bit of general tidying, ensuring the resources are pumped up so that actually when you want to go out and use them, you can actually use them rather than getting there and all the basketballs are flat. So it’s kept in a decent order.” (Participant 10, Class Teacher, school FSM% 9.8, IMD 10)*

### Theme 3: logistical challenges undermine the potential of the facilities and resources

Logistical challenges consistently came through as a barrier to using and maximising the potential of the facilities and resources a school has or has access to. A school may have spaces or equipment for activity, but the use of these competed against other school priorities, and so the existence of them alone was not enough to ensure PE and wider pupil physical activity. Timetabling of school indoor space specifically was frequently mentioned. Participants described a number of uses for indoor halls, such as pupil assemblies, rehearsals for school plays, as dining halls for lunch, and as spaces used by wrap-around childcare before and after school. Scheduling these facilities can be very challenging for schools and undermined their potential to be used for pupil physical activity.

*“[The PE teacher] timetables it so different year groups are doing gym [in the hall] at different points of the year. […But] every now and again there's a school performance that needs the hall on a Wednesday afternoon and that’s your PE slot.” (Participant 10, Class Teacher, school FSM% 9.8, IMD 10)*

Lunch and break times were often described as too short to warrant getting out equipment for pupils to use, and transitions between classroom-based lessons and PE or movement breaks, especially if children had to change clothes, further eroded the time for engagement in physical activity. Class teachers who teach PE described being unable to prepare a space or equipment for a lesson in advance because the space would be in use until the moment their class began. Teachers have to transition from their classroom to the PE space with pupils, and therefore often rely on using equipment that is minimal and quick to set up in order to manage class behaviour. This issue has been compounded by staffing pressures in schools, where previous capacity for Teaching Assistants (TAs) to set up for a PE lesson, take a class for a movement break, or support break time activities is being lost (Theme 1).

*“[break time is] such a short session so we don’t get a lot [of equipment] out because it’s only 15 minutes. By the time you’ve got it out, it’s time to put it back in again.” (Participant 7, Deputy Head, school FSM% 15.3, IMD 4)*

*“This year, there’s a new set-up where the [TAs] aren’t really per classroom. They’re spread out. […] I think that does impact things like Daily Mile [a movement break] and smaller bits of activities in the day. Because the TA would do that. They’d take them out while the teacher could sort something else out and now they can’t do that anymore.” (Participant 4, Teaching Assistant, school FSM% 48.0, IMD 1)*

### Theme 4: the potential of facilities and resources change throughout the school year

This theme establishes how the potential of facilities and resources to support pupil physical activity changes across the school year. Although timetabling of facilities and other logistical issues are always a challenge in school and impact on pupil physical activity (Theme 3), the extent of this changes with the seasons.

Schools described outdoor spaces that were unusable for several months of the year usually due to wet weather making fields muddy or MUGAs/sports courts slippery. Exacerbating this, in these seasons staff avoided pupils going outside in the rain as pupils often do not have appropriate clothing for the weather, which impacts PE, break times, and active clubs. This was described as the downside of an active uniform, or children coming dressed for PE on PE days, as they had no change of clothes if they got wet during a PE lesson. These factors increased the pressure on indoor facilities that are already maximised (Theme 3), and in turn school staff described cancelling PE lessons or attempting to do them in classrooms or other inappropriate spaces.

*“If your PE session is at 10:00 in the morning and it's drizzly and raining, then children have got to come back inside for the rest of the day and sit in damp clothes. Because at this school […] when it’s your PE day, you come in dressed in your PE kit. […] The drawback in that is, obviously, that’s their kit for the rest of the day, so if they get wet, there’s no backup clothing to change into.” (Participant 10, Class Teacher, school FSM% 9.8, IMD 10)*

*“so it’s rained for God knows how long, so then you can’t get out, or the court is flooded, or whatever. […] if that happens, then there are two options. One, we’ll put on a yoga video, or we’ll just do something else. And then the PE doesn’t get rescheduled, because those [indoor] spaces are so packed.” (Participant 14, PE Lead, school FSM% 20.9, IMD 4)*

However, there are also seasonal moments in a school year when facilities are under increased pressure due to other school activities which are prioritised, which further undermines the potential of facilities and resources to support pupil activity (Themes 2 and 3). Times mentioned included the weeks before Christmas and the final summer term when school halls are often used for rehearsals for school plays or other seasonal activities, or when halls are needed for pupils to sit school exams. When these periods of additional priorities combine with poor weather the pressure on facilities is increased.

*“we’ve got the Nativity that need the indoor hall for their practice, and things like that, we have various things going on, like, I don’t know, a space dome is coming in, they need the hall. So PE often will have to get moved for those things, and if it gets moved, it just doesn’t get done” (Participant 14, PE Lead, school FSM% 20.9, IMD 4)*

### Theme 5: facilities and resources are not always fit for purpose

School staff described how physical activity facilities and resources are not always fit for purpose. Quality was described as an issue, such as old or broken equipment. This of course can be solved through simply replacing or increasing these resources (Theme 1), and has been touched upon already in terms of the maintenance of facilities (Theme 2) and the seasonality of facilities such as fields (Theme 4).

However, schools elaborated on the issue of how fit for purpose both outdoor and indoor facilities were, beyond issues that could be solved by replacing or upgrading, demonstrating that the presence of a facility does not always mean it is suitable for physical activity. Fields were described as bumpy or sloped (not just seasonally) so not suitable for sports day or athletics. The design and location of indoor halls were also an issue for some schools, where some had features that inhibited sport and activity, and others were located with classrooms leading onto the hall, which was disruptive both for pupils in classroom lessons and pupils doing PE in the hall.

*“Inside the building, we have got two lovely big halls, but the classrooms […] all feed off of the hall, […]. It is really difficult getting the children's attention because there’s so much stuff going on, there are people walking through all the time, and there’s people having conversations. […] The acoustics in the hall are awful, so things like that are barriers.” (Participant 2, PE Lead, school FSM% 31.6, IMD 2)*

*“[Our hall] is a reasonable size. But there are two massive pillars in the middle of it, because they're holding up this- They're key for the structural support of the [building] above. Most visitors wonder how I teach PE in there, because it looks quite strange.” (Participant 15, Dedicated PE Teacher, school FSM% 18.3, IMD 6)*

Issues of how fit for purpose physical activity resources were also came through in regards to the external provision of PE and sport coaches, PE curriculum resources, and external schemes for PE and physical activity. Many primary schools buy in private external companies to provide PE, lunch time activities, and staff continuing professional development (CPD). Participants described experiencing great variety in the quality of these external coaches, who were seen as highly skilled in teaching sport, but at times lacking the broader skills needed to manage emotional wellbeing, behaviour, and diverse pupil needs.

*“Some sports coaches are brilliant. Some of them are not, particularly when you have a class with more complex needs and you need to have that experience of working with lots of different children.” (Participant 32, Deputy Headteacher, school FSM% 9.7, IMD 3)*

Participants described how PE curriculum resources that a school bought in, or external schemes for PE and physical activity such as the Daily Mile, are often not adaptable or suitable to their particular school context. These schemes at times made assumptions about the facilities a school could access, the amount of equipment they would have, or the length of time that schools give to a session (Theme 3).

*“we find with lots of [physical activity schemes] they’re catered towards large schools, and do not work for us at all.” (Participant 33, PE Lead, school FSM% 7.1, IMD 7)*

*“I brainstormed so many ways, thinking, ‘How could we get that [The Daily Mile] to work in our school?’ it’s just a physical impossibility.” (Participant 15, Dedicated PE Teacher, school FSM% 18.3, IMD 6)*

## Discussion

In this analysis we explored primary school staff perspectives of the role of school facilities and resources in pupil physical activity in the school day. From their experience within schools, it was clear that staff felt that facilities and resources created a potential to support physical activity, but that simply having the presence (or absence) of, for example, an indoor hall, playing field, or particular equipment, was not enough to ensure they were used to maximise pupil activity. Rather, it is important to understand the way in which these facilities and resources are used, managed, or engaged with in order for individual strategies targeted to a school to be developed that lead to meaningful changes in children’s physical activity in primary school.

We argue that facilities and resources create potential for pupils to be physically active, but that this potential can be undermined or maximised by the social context of the school, logistical challenges and solutions, seasonal variation in the weather and school activities, and how fit for purpose a school’s facilities and resources are. These findings highlight that the relationship between the school environment and physical activity is complex and nuanced. This present study therefore builds on previous work that has sought to identify key features of school environments [30–37]. The findings of this literature have been mixed in regard to individual features of the school environment and their role in pupil physical activity, and generally conclude that broader factors such as quality, content, staff, or policies are important and need to be understood. This paper contributes to this gap to provide staff insight into how facilities *and* resources are managed, used, and engaged with in the day-to-day of delivering Physical Education and physical activity in primary schools, providing some explanation as to why the presence of facilities alone does not necessarily increase pupil physical activity. Changes in a school’s facilities and resources alone will not necessarily be enough to increase pupil physical activity. This has implications for policy which focuses on provision alone, without consideration of school contextual factors.

These findings reflect the socioecological model of health that emphasises how health is influenced by the interplay between individuals and multiple layers of their environment, including interpersonal relationships, communities, societal structures, and policies, highlighting the need to address factors at all levels to create comprehensive and sustainable health improvements [52]. Our results suggest that while facilities and resources create the potential for physical activity, it is vital that social, organisational, and logistical factors are considered in conjunction with facilities and resources to ensure physical activity can be effectively and sustainably promoted. This also emphasises the importance and need for whole-school approaches to physical activity [18] that integrate physical activity into school culture, so that physical activity and facilities are used to their maximum potential.

Existing evidence suggests that staff play a significant role in pupil physical activity at school, via senior leaders setting priorities or policy, and other members of staff with passion or motivation to support pupils to be active (Walker, et al.: The complexity of promoting physical activity in English state primary schools: an in-depth qualitative analysis of the role of social context, Under review). Conversely, primary school teachers can lack confidence and experience in delivering PE lessons ((Walker, et al.: The complexity of promoting physical activity in English state primary schools: an in-depth qualitative analysis of the role of social context, Under review), [53]). The findings of this study align with this, whereby we argue that this social context can act as both a barrier and facilitator to engaging with school facilities. Literature has suggested there is a relationship between school policy and the physical environment in pupil physical activity that must be understood [34]. This present study sheds light on the dynamics of this relationship by highlighting several ways in which school leaders can make decisions that undermine or maximise the potential of the built environment to support pupil physical activity. Furthermore, previous literature exploring school facilities and resources has not, to our knowledge, highlighted the extent to which timetabling and logistical issues are a key factor in whether or not school facilities are used for physical activity. This study demonstrates the extent to which logistical issues shape school life and schools’ ability to ringfence and deliver opportunities for pupils to be physically active. Interestingly, the role of indoor space for physical activity may play a larger role than has been recognised in literature, which has focused on outdoor space and its potential to support physical activity [30, 31, 33], with some studies and reviews focussing on classroom settings [54, 55]. This may be particularly relevant to UK settings or other countries with wetter climates and relatively limited space in urban environments. Logistical challenges may be further compounded by what staff have reported as decreasing budgets (Walker, et al.: The complexity of promoting physical activity in English state primary schools: an in-depth qualitative analysis of the role of social context, Under review). Policy efforts should include funding for ongoing maintenance, staff training to maximise facility use, and adaptable programs that account for the diversity of school contexts.

Use of facilities and resources changes over the school year. Previous research has established clear evidence on the seasonality of physical activity for both children [56] and adults [57]. Although the weather has been identified as a key factor in determining the amount or type of physical activity pupils engage in [53, 58, 59], this literature has not explored in depth how this seasonality plays out in schools. This current paper contributes to this literature by offering insight into how and why seasonality influences children’s physical activity in schools. Some facilities are under increased pressure at different times of year due to adverse weather conditions impacting upon the use of outdoor facilities, but, significantly, also due to broader seasonal school activities and priorities. This is important as it suggests that the focus on the weather is overly simplistic and other factors, that are within the control of the school, are at least as important. While seasonal weather constraints are unmodifiable, schools could mitigate these challenges through policies that prioritise indoor space usage during adverse weather. Future interventions could explore solutions for modifiable factors, such as revisiting indoor space usage policies during peak activity periods.

Lastly, this paper highlights how facilities and resources are often not fit for purpose to support pupil physical activity, likely linking to staff-reported budget and funding challenges in schools (Walker, et al.: The complexity of promoting physical activity in English state primary schools: an in-depth qualitative analysis of the role of social context, Under review). This evidence may explain why moving beyond studies that simply audit the presence or absence of features in a school environment is necessary to understand the associations between these features and children’s activity levels. It also suggests that any intervention to support pupils to be active should begin by understanding the particular context and state of play of physical activity in that school, and then be adaptable and sensitive to school context to ensure it is meeting a specific schools’ needs.

Our findings suggest a need for future research that understands schools, and physical activity interventions in them, contextually, taking account of the nuance of how facilities and resources are used, managed, and engaged with within a school system throughout the school year. This would warrant future qualitative research to explore the findings suggested here in more depth. It also suggests that current approaches to audit-based measurement tools could be improved by including measures that might capture not just the presence of facilities and opportunities, but staff perceptions of their quality, the school culture, or school readiness to change, which could then help inform future intervention development.

### Implications for policy and practice

This study suggests that physical activity interventions and policy would benefit from moving beyond the first step of creating opportunities or increasing facilities to support pupils’ physical activity in schools, to ensuring a focus on how these will be maintained and engaged with and embedded in school culture. It is also essential that any changes to school physical activity infrastructure is appropriate to that school’s specific need and context, and that resources and schemes are designed to be adaptable for schools. School leaders could consider forming committees to periodically evaluate the functionality of their physical activity spaces and propose cost-effective adaptations tailored to their specific needs. Key findings and implications can be seen in Table 4.

**Table 4:** Key findings and implications

Key finding	Practical implication
The existence (or not) of facilities and resources alone is not enough to determine pupil physical activity.	Physical activity interventions and policy need to move beyond simple audits of the existence of facilities and resources and creating opportunities.
A social context conductive to physical activity is important to maximise potential of physical activity facilities and resources.	Physical activity needs to be embedded within school culture to maximise use and potential of facilities and resources. Staff training will likely play a key element in this.
Logistical challenges undermine use of facilities and resources.	Policy efforts should include funding to ensure that staff resources and other logistical challenges are minimised.
Facilities and resource potential varies throughout the school year	Physical activity interventions and policy efforts should consider how facilities, resources and opportunities vary throughout the school years, so that physical activity can be maintained over the school year.
Facilities and resources are not always fit for purpose	It is vital that physical activity interventions consider the quality of facilities and resources. Policy efforts should include funding for ongoing maintenance of facilities as their quality may reduce over time.

### Strengths and limitations

Our study’s strength lies in the sample of participants and schools included. The participants are diverse in terms of the positions they hold in schools, and importantly the contexts of the schools in which the participants work are geographically, socioeconomically, and ethnically diverse, as well as a variety of school sizes. This diversity in the sample gives the data a balance of perspectives and experiences and strengthens the arguments and claims this paper makes about school contextual factors and differences. The main limitation of this study, however, is that it was evident in the analysis that school context is complex and changes throughout a year. The semi-structured interview method, while providing a broad range of perspectives, remains relatively shallow for exploring the issue, and in-depth methods and approaches would be of value. In addition, interviews may not capture longitudinal shifts in school practices or non-verbal barriers observed in real-time, as was suggested to be important within our findings. Furthermore, despite intentional recruitment tracking and processes, it is possible that more physically active participants and schools may have participated, due to valuing physical activity or avoiding scrutiny if physical activity was not prioritised, potentially skewing the sample toward higher-performing schools in this area. However, as physical activity levels are linked to both ethnicity and socioeconomic status [11], our inclusion of schools from these contexts may have alleviated this issue.

## Conclusion

Findings suggest that facilities alone are insufficient to guarantee physical activity; however, this observation reflects the experiences of schools in the Bristol area and may not generalise to other contexts. Factors such as the social context of the school, logistical and timetabling challenges, seasonality, and how fit for purpose facilities are, are important factors in school staff and pupils’ use and management of these facilities, and whether their potential is maximised or undermined. Physical activity interventions and policy would benefit from moving beyond the first step of auditing built environments, creating opportunities, or increasing facilities to support pupils’ physical activity in schools, to ensuring a focus on how these will be maintained and engaged with in each school specific context.

## Supplementary Information

Supplementary Material 1.

Supplementary Material 2.

## Data Availability

As the PASSPORT project is still ongoing, data are not currently available. At the end of the project, data will be published as a restricted access dataset on the University of Bristol’s data repository (https://data.bris.ac.uk/data/) and access granted to approved researchers on request.
