# Supplementary material for: “We just have to work with what we’ve got”: a qualitative analysis of contextual challenges in facilities and resources for pupil physical activity in English primary schools
Source: BMC Public Health. 2025 Feb 21;25:726. doi: 10.1186/s12889-025-21895-1 (PMC11846469; doi:10.1186/s12889-025-21895-1)
Supplement: Supplementary file 1 — Supplementary Material 1. [file 12889_2025_21895_MOESM1_ESM.docx]

**School staff and community interview guide:**

**Factors influencing physical activity**

This interview is part of a larger project called PASSPORT. In this project we are hoping to identify what aspects of school are important in pupil physical activity. We the plan to provide schools with a way to assess their schools, identify any areas that can be improved, and develop bespoke programmes to support pupil activity.

In this interview we want to talk about and identify the aspects of school that are important in pupil physical activity.

*Confirm they have read the participant information sheet and have completed the consent form.* ***Reconfirm consent on the audio recording.***

1. **Warm up (just a few mins)**

- Can you tell us a bit about your role in the school? How long have you been at the school? How long have you worked in the primary education sector?
- Can you tell us a little about your school? Its setting, its community, its ethos?

1. **Physical activity at your school**

*When we talk about physical activity, we are talking about kids being generally active and moving. For example walking to school, running around at break times, during classes, PE lessons, and active clubs.*

**General physical activity**

- What do you feel makes an active school?
  - What are its key features?
    - An active culture?
    - Family support?
  - What would be needed for these to become a reality?
  - What are the real-world challenges related to this?
    - Academic pressures?
    - Senior Leadership Team buy in?
    - Space/facilities?
    - Funding/budget?

**Active travel**

- Roughly what percentage of children at your school school normally use active modes of travel to school?
  - What do you think your school does well in encouraging active travel? Schemes?
- What do you think schools need in order to support active travel?
  - An active travel policy?
  - Facilities?
  - Culture?
  - Role of school staff?
  - Role of parents?
  - Local environment?
- What are the real-life challenges to children actively traveling at your school?
  - Safety?
  - Distance?
  - Parental factors – travel to work, time, etc
  - How could we help to address these challenges?

**Break and lunch times**

- How would you describe pupils’ physical activity at break and lunch times?
- What do you think a school needs to support or encourage physical activity at break and lunch time?
  - Equipment?
  - Space?
  - Staffing?
- What are the real-life challenges to getting children physically active during breaks/lunch time at your school?
  - Children not interested?
  - Gender differences?
  - Rota systems or restrictions on space?
- How could we help to address these challenges?

**Before and after school**

- Does your school provide wrap around care before and after school (e.g. a breakfast club and after school childcare club)?
  - If so, do these have any physical activities on offer?
- How would you describe the active club provision at your school?
  - Wide variety? Every day?
  - Are these well attended?
  - Who provides them? Why?
- What do you think your school does well in relation to their active clubs?
- What do you think a school needs to create successful active clubs?
  - Staff volunteers?
  - Low cost to parents and school/funding?
- What do you feel are the real life challenges to this?
  - Staff time?
  - Facilities?
  - Cost to parents?
  - Breadth of offer?
- How could we help to address these challenges?

**Physical Education (PE)**

- How would you describe PE at your school?
- What do you think your school does well in terms of their PE programme?
  - Do you follow an external programme? Why? What do you think of it?
- What is needed for a successful PE programme?
  - Role of PE policy?
- What are the real-world challenges to this?
  - Curriculum pressures
  - Teacher training
  - SLT support
  - School ethos
  - Facilities – is PE ever cancelled due to space?
- How could we help to address these challenges?

**During non-PE lessons**

- Does your school promote active learning or movement breaks?
- Do you think they are useful in promoting activity and meeting learning objectives?
- What would be needed to support more active learning/movement breaks?
  - Resources?
  - Reduced pressures?
- What are the real-life challenges to promoting active learning/movement breaks in your school?

**Depending on interviewees role, we’d like to ask about budget and spending to support physical activity**

- Do you feel there is sufficient budget or resource to deliver what you’d like to?
- How do you currently spend your PE and Sports Premium?
  - General type of activity or area e.g. maintenance of facilities, equipment, external provision
  - Any targeted groups e.g. swimming for children not meeting curriculum requirement
- How are these decisions on how to spend the PE and Sports Premium made?
- How do you find the process of receiving, spending, and reporting on the PE and Sports Premium?
- Do you use Pupil Premium to support physical activity?
- Do you use any other funding streams?

1. **Final thoughts**

- Thinking about everything we have discussed, in your specific school, what do you think are the main factors that shape how active your pupils are?
- Is there anything about your school or community specifically that needs to be considered when thinking about physical activity?
  - The pupils and their needs?
  - Any issues around staff and staffing? Training?
  - The space and facilities you have?
  - The culture/ethos and strategic priorities?
  - Your current strengths?
- Reflecting on the discussion, is there anything you’d like to comment on regarding the factors that a school is able to change or influence, and those a school cannot?
- Anything else you would like to add about anything we have discussed today?

*Thank participant for their time and end interview.*
