# Supplementary material for: “We just have to work with what we’ve got”: a qualitative analysis of contextual challenges in facilities and resources for pupil physical activity in English primary schools
Source: BMC Public Health. 2025 Feb 21;25:726. doi: 10.1186/s12889-025-21895-1 (PMC11846469; doi:10.1186/s12889-025-21895-1)
Supplement: Supplementary file 2 — Supplementary Material 2. [file 12889_2025_21895_MOESM2_ESM.docx]

**School staff and community interview guide:**

**Exploring tools to support physical activity provision**

This interview is part of a larger project called PASSPORT. In this project we are hoping to identify what aspects of school are important in pupil physical activity. With this, we will provide schools with a way to assess their schools, identify any areas that can be improved, and develop bespoke programmes to support pupil's activity.

In this interview we want to talk about your thoughts about our research plans and using two tools that we are planning to develop to help make decisions about physical activity provision in your school. We want to know if you think these tools could be useful, and how they might work. We also want to talk about how we can test this approach in a way that works best for schools.

*Confirm they have read the participant information sheet and have completed the consent form.* ***Reconfirm consent on the audio recording.***

1. **Warm up (just a few mins)**

- Can you tell us a bit about your role in the school? How long have you been at the school? How long have you worked in the primary education sector?
- Can you tell us a little about your school? Its setting, its community, its ethos?

1. **A new approach to physical activity programmes in schools**

*We want to design two tools. The first is for schools to use to help them identify their specific physical activity needs and evaluate what they already have and do well to support physical activity. The second would then be used by a school to select additional physical activity program elements from a list of options, that will work for your school and your community.*

*These activities could include enhancing the offer of active clubs, staff CPD, improving active travel infrastructure, and others.*

- What are your initial thoughts on this approach?
  - Would it be helpful? Welcome? Don’t see a need for it?
- How do you think other/broader school staff would respond to this approach?
- What would this approach need in order to be successful in a real-world scenario?
  - Senior Leadership Team buy in, all staff buy in?
  - Prioritisation?
  - Focused in the School Development Plan?
- Have you experienced something similar to this in the past?
  - If so, what were the successes or challenges to this programme?

1. **The context assessment tool**

*This will be a tool to assess the physical activity opportunities a school currently provides, as well as other important factors at that school such as available space, school culture, and the school community. This will be based on information school staff have already given us about pupil physical activity at school.*

- What would a school need to be able to use a tool like this?
  - Level of external support? How – email communication, in person meetings?
  - Training?
- Which school staff would need to be involved in using this tool?
  - Is it realistic that those members of staff could be involved?
  - What would be needed to help them be involved?
  - Do you feel children could be involved? How might we include them? School Council?
- How much time could schools realistically give to this?
  - Would you be happy to use the tool more than once, e.g. each year?
- What would be the best format for this kind of tool?
  - A paper survey
  - An online tool
  - An app

1. **Selecting additional activities to support pupil physical activity**

*Once the context assessment has been completed, the next step would be to use a second tool to choose from a range of options that would work within your specific school to support pupil physical activity, complement what you already have and do, and work for your community.*

- - Who would need to be involved in selecting the options/using the tool for the school?
    - PE Coordinator/Lead? Senior Leadership Team?
    - Do you feel children should be involved? If so, how?
  - What information would be needed to be able to select activities?
    - What level of detail would be useful for each option?
    - Where might it fit in the school day?
    - How the activity is thought to support physical activity, and by how much? (i.e. increase motivation, physical literacy, cycling confidence, etc.)
    - Whether staff training is required to deliver the activity?
    - Who would be involved in delivering the activity? I.e. external agency
  - Do you have any thoughts on what format would be best for this tool?
    - A booklet, form, paper
    - An online tool
    - An app
  - How much external support would a school need to complete this?
    - An external person to help complete the tool and select options?
    - Training?
  - How much time could schools realistically give to using this tool?
  - How much preparation / lead time do you think would be needed to set up the activities the school selects? I.e. between using the tool and delivering on what it recommends
    - What factors might affect this prep time?
    - How long has this taken your school to set up programmes in the past? (i.e. PE and Sport premium spending, organising external organisations to come into school)

1. **Reflecting on this approach**

- Having now explored the idea of these tools in detail, what are your thoughts on this approach? Have they changed since the start of the conversation?
  - Would it be helpful? Welcome? Don’t see a need for it?
- Would these tools support your decision making around physical activity offer and spend?
- How might this approach work within the broader aims and priorities of the school?
- Some of the options may include changes within the school or be delivered by school staff or external agencies. Are there any practical considerations about either of these we should consider?
  - Policies, funding, admin etc?
  - The need for extra planning/prep time?
  - Would this influence the activities you would choose?
- How do you see these tools and physical activity options working alongside any current awards or programmes you have in your school?
  - E.g. Bristol Healthy Schools, MODESHIFT Stars etc.
- What would be needed to make this approach – the context assessment and selecting new activities - sustainable beyond any involvement from the University?

1. **Testing out the approach**

*In the PASSPORT study, once we have developed these tools, we are going to pilot them, including delivering the activities the school chooses, with a small number of schools. Following that we will trial the whole approach through a research study in more schools and measure children’s physical activity. We have money to give schools in both the pilot and the main trial to fund the new activities and compensate schools for the inconvenience of the research.*

**School and pupil recruitment**

- Do you have any thoughts on how we can recruit schools in more disadvantaged neighbourhoods to take part?
- How could we promote whole school buy-in?
- What is the best way to encourage children and their families to sign up to taking part?
  - And keep them engaged over a year or more?
- How do we encourage and support children and families who aren’t normally engaged in physical activity to take part in the research?
- How do we support different children with different ability levels to participate? Children with mobility issues?

**Data collection**

*When we test the approach, schools would be asked to use the tools to assess what they currently do, and then select their bespoke physical activity program. We would then go to a school to recruit and collect activity data from pupils using wrist-worn accelerometers, or activity devices on several occasions.*

*To make the trial work, a few aspects would be really important:*

1. *We would need to collect data from pupils, and access them via the school, at 5 points. We would need a school to commit to all of these measurement points. These measurement points would be spaced throughout the year when pupils are in Year 5, with the first time point potentially in term 5 or 6 of Year 4. A school would know the weeks for each time point shortly after they agree to take part. Each time would involve an initial recruitment session, then researchers coming to the school to hand out and collect wrist worn devices to the pupils whose parents had signed them up to the research.*
   - What would be the real-world challenges to doing this? Does this sound feasible?
   - What if we did measurements and came into school on 4 rather than 5 occasions?
   - What do we need to consider with the spacing out of these across the year? Does one every two terms work? Or 8-10 weeks apart? There would never be more than one in a term.
   - How can we make this as easy for schools as possible?
   - Is there anything we need to consider if the measurements span years 4 and 5?
2. *The time in the school year (which term) at which the school begins its new/additional activity programme will be randomly selected. Schools will be told when they will get the new activity programme when they sign up to the study and can start on any set-up / prep work, but they can’t change when the new programme takes place.*
   - What would be the real-world challenges to doing this?
   - What do we need to consider about school decision and planning time frames?

*Thank participant for their time and end interview.*
